# Facing financial barriers to healthcare: patient-informed adaptation of a conceptual framework for adults with a history of cancer

**DOI:** 10.3389/fpsyg.2023.1178517

**Published:** 2023-05-15

**Authors:** Caitlin B. Biddell, Austin R. Waters, Rebekah S. M. Angove, Kathleen D. Gallagher, Donald L. Rosenstein, Lisa P. Spees, Erin E. Kent, Arrianna Marie Planey, Stephanie B. Wheeler

**Affiliations:** ^1^Department of Health Policy and Management, Gillings School of Global Public Health, University of North Carolina at Chapel Hill, Chapel Hill, NC, United States; ^2^Lineberger Comprehensive Cancer Center, University of North Carolina at Chapel Hill, Chapel Hill, NC, United States; ^3^Patient Insight Institute, Patient Advocate Foundation, Hampton, VA, United States; ^4^Departments of Psychiatry and Medicine, University of North Carolina at Chapel Hill School of Medicine, Chapel Hill, NC, United States; ^5^Cecil G. Sheps Center for Health Services Research, University of North Carolina at Chapel Hill, Chapel Hill, NC, United States

**Keywords:** cancer, financial toxicity, financial burden, access to care, financial barriers

## Abstract

**Background:**

Cancer-related financial hardship is associated with negative clinical outcomes, which may be partially explained by cost-related delayed or forgone care in response to financial barriers. We sought to understand patient experiences facing financial barriers to medical care following a cancer diagnosis.

**Methods:**

We conducted virtual, semi-structured interviews in Fall 2022 with 20 adults with a history of cancer who had experienced cancer-related financial hardship in the prior year. We used template analysis within a pragmatic paradigm, combining constructivist and critical realist theoretical perspectives, to analyze interview transcripts and adapt an existing conceptual framework of financial barriers to care.

**Results:**

The majority of interviewees identified as women (70%), non-Hispanic white (60%), and reported an annual household income of <$48,000 (60%). As interviewees sought to overcome financial barriers, they described substantial frustration at the limitations and complexities of United States health and social care systems, resulting in a reliance on a fragmented, uncertain resource landscape. The administrative burden resulting from bureaucratic systems and the advocacy responsibilities required to navigate them ultimately fell on interviewees and their caregivers. Thus, participants described their ability to overcome financial barriers as being influenced by individual and interpersonal factors, such as social support, comfort asking for help, time, prior experience navigating resources, and physical and mental health. However, participants noted health system organizational factors, such as whether all new patients proactively met with a social worker or financial navigator, as having the potential to lessen the administrative and financial burden experienced.

**Conclusion:**

We present an adapted conceptual framework outlining multi-level factors influencing patient experiences coping with financial barriers to medical care. In addition to influencing whether a patient ultimately delays or forgoes care due to cost, financial barriers also have the potential to independently affect patient mental, physical, and financial health.

## 1. Introduction

A cancer diagnosis and subsequent treatment in the United States can impose substantial costs, both medical (i.e., out-of-pocket costs) and non-medical (e.g., transportation, lost income), on patients and their families. As a result, it is estimated that almost half of individuals with a history of cancer experience financial hardship, including material conditions, psychological response, and coping behaviors (Altice et al., 2017; Zheng et al., 2019; Jiang et al., 2022). More specifically, 70% of adults ages 18–49 with a history of cancer report one or more domains of financial hardship, followed by 63.2% of those ages 50–64, and 38.7% of those 65 years and older (Zheng et al., 2019). As such, “financial toxicity” has emerged as a term over the past decade to relate the financial consequences of cancer treatment to other treatment toxicities routinely monitored and addressed (Zafar and Abernethy, 2013).

Cancer-related costs, compounded by underlying financial vulnerability, may lead patients to experience financial barriers to accessing and paying for medical care during active treatment and into survivorship. Almost 20% of adults with a history of cancer report forgoing medical care and/or prescription medications due to cost (Weaver et al., 2010). Financial access barriers may lead to missed appointments (Maldonado et al., 2021), treatment and medication nonadherence or delays (Knight et al., 2018), and forgone surveillance and preventive care. Furthermore, the burdens of cost-related care interference are not experienced equally, with a higher prevalence among uninsured or publicly insured patients (al Rowas et al., 2017; Amin et al., 2021), patients of color (Weaver et al., 2010; Wheeler et al., 2018), and low-income patients (Amin et al., 2021). As such, understanding and addressing patient financial hardship, and how such hardship influences access to care (Levesque et al., 2013), is a necessary step toward promoting equitable cancer care delivery (Tucker-Seeley, 2023).

Conceptual frameworks describing the impact of financial hardship on patients with cancer have identified delayed and forgone care as a coping behavior to reduce costs (Altice et al., 2017; Jones et al., 2020); however, conceptual clarity surrounding cost-related care interference is lacking. Though not developed among patients with cancer, Campbell and colleagues developed a conceptual framework of the role of financial barriers to healthcare in contributing to health outcomes among patients with cardiovascular-related chronic disease in Canada (Campbell et al., 2016). This framework, developed using grounded theory, conceptualizes both the causes of perceived financial barriers and the factors influencing the extent to which perceived financial barriers to healthcare translate into care avoidance, adverse healthcare events, and negative clinical outcomes (Campbell et al., 2016).

Given notable differences between a cancer diagnosis and chronic cardiovascular conditions, as well as the Canadian versus United States healthcare systems, there is a need to adapt and update this model to reflect patient experiences with financial barriers to healthcare following a cancer diagnosis in the United States. The United States healthcare system does not provide universal healthcare coverage and consists of both public and private payers. Private health insurance coverage is most commonly obtained through employers, public Medicaid coverage is provided to low-income individuals meeting eligibility criteria through states, and public Medicare coverage is provided to individuals with disabilities and adults over 65 years of age through the federal government.

This study builds off of Campbell and colleagues’ conceptual framework of financial barriers, as well as prior qualitative analyses documenting cancer-related financial hardship, to understand the experiences of patients with cancer facing financial barriers to healthcare in the United States (Amir et al., 2012; Timmons et al., 2013; Schröder et al., 2020). Using the conceptual framework of financial barriers as a guide, we specifically probed on patient perceptions of factors influencing the extent to which perceived financial barriers resulted in delayed and forgone medical care. Ultimately, a better understanding of how patients experience financial barriers to care serves to inform patient-centered approaches to reducing cost-related cancer outcome disparities.

## 2. Materials and methods

### 2.1. Study design/research approach

In order to capture in-depth patient experiences, we conducted qualitative interviews with individuals with a history of cancer living in the United States. We then conducted a qualitative analysis using a hybrid inductive and deductive template analysis approach (King and Brooks, 2017; King et al., 2018). Template analysis is an established qualitative thematic analysis approach involving the iterative development of a coding template and subsequent thematic interpretation that can be used in the context of a range of qualitative paradigms (King and Brooks, 2017; King et al., 2018). Our overarching approach to this research study was pragmatism, which involves the combination of approaches for the purposes of understanding a given research problem (Moon and Blackman, 2014). The first stage of analysis was largely inductive, and we approached this phase with a constructivist theoretical perspective, focusing on meaning-making from participant lived experiences within their social environments (Moon and Blackman, 2014). Given that there is an existing conceptual framework of patient experiences facing financial barriers to healthcare developed using grounded theory (Campbell et al., 2016), we then layered on this framework deductively as a way of situating the knowledge generated through participant experiences into the existing body of knowledge on this topic. Lastly, in line with a critical realist qualitative paradigm, we structured the meaning gleaned from our qualitative inquiry into an adapted conceptual framework intended for further use and revision. The critical realist paradigm allowed this conceptualization to inform our codebook development and thematic interpretation. Themes resulting from the hybrid inductive/deductive template analysis then informed the adaptation of this conceptual framework for adults with a history of cancer. A critical realist approach is particularly well suited to health services research in that it seeks to recognize and acknowledge objective health outcomes while remaining open to variation in how participants experience and understand those outcomes (Ritchie et al., 1994; Archer et al., 1998; Clark et al., 2007; King et al., 2018).

### 2.2. Participants

Potential interview participants were identified through the Patient Advocate Foundation (PAF), a national non-profit organization providing financial assistance and social needs navigation services to patients with serious and chronic illness. Individuals were considered eligible if they met the following criteria: (1) diagnosed with cancer (any site), or received active cancer treatment, in the prior one to 5 years, (2) age 18 or older at the time of diagnosis, (3) completed a survey administered in English by PAF in May 2022 and indicated willingness to be contacted for future research, and (4) experienced cancer-related financial hardship in the past 365 days (self-reported via screener questionnaire). Patients receiving the PAF survey received assistance from PAF between July and December 2020.

A total of 218 individuals met the first three eligibility criteria based on data collected from the PAF survey. From this subset, PAF emailed a screener questionnaire to waves of purposively sampled individuals in order to maximize diversity with regard to age, race, ethnicity, gender, and sexual orientation. Of the 218 eligible individuals, 111 were emailed. Potential participants were considered to have experienced cancer-related financial hardship if they self-reported experiencing difficulty paying for medical care or prescription medications, reducing spending on basic necessities (e.g., food, housing) to get needed medical care or prescription medications, or delaying or forgoing medical care because it cost too much in the past 365 days. Eligible individuals were then emailed by a member of the study team (CB) to schedule an interview. Participants were also given the option to schedule by phone. Additionally, two participants, who also met all eligibility criteria, were referred to the study via snowball sampling. Participants were compensated for their time with a $25 electronic gift card. The institutional review board approved this study (UNC-CH IRB#22-0467).

### 2.3. Data collection

A member of the research team (CB) conducted virtual, audio-only semi-structured interviews between August and November 2022. Interview questions were guided by an in-depth, semi-structured interview guide, which was informed by Jones and colleagues’ conceptual framework of financial burden in adult cancer survivors (Jones et al., 2020) and Campbell and colleagues’ conceptual framework of financial barriers to care in adults with chronic cardiovascular conditions (Campbell et al., 2016). The guide was refined through pilot testing with three patient advocates recruited from PAF’s Patient Insight Institute Experts by Experience Advisory Committee. The complete interview guide is included in Supplemental Appendix 1.

Interviews were conducted until thematic saturation was achieved in relation to the primary research questions (Malterud et al., 2016; Saunders et al., 2018). All interviews were audio-recorded, transcribed using an online transcription tool and then cleaned and quality checked against audio files. Sociodemographic information for interview participants was collected in the electronic survey administered by PAF in May 2022 and included: age category, race, ethnicity, gender, sexual orientation, education, employment status, marital status, household income category, self-described rurality, health insurance status, cancer type, and time since diagnosis.

### 2.4. Data analysis

To maximize reflexivity, the template analysis took place concurrently with data collection. The research team first engaged in data immersion, or familiarization, by writing analytic memos following each interview and developing a qualitative matrix organized by participant and interview domain (Miles et al., 2015). This matrix included key patient characteristics hypothesized to influence the experience of financial barriers to care.

Second, two independent coders (CB, AW) analyzed interview transcripts in Dedoose version 9.0.62 (SocioCultural Research Consultants, LLC Dedoose, 2022); using a codebook developed via a hybrid inductive and deductive approach (Saldaña, 2013; Miles et al., 2015). In first cycle coding, CB conducted open coding on 20% of the interviews (*n* = 4), during which transcript segments were categorized based on emergent ideas, both descriptive and thematic. Codes resulting from open coding were condensed into a coding scheme by combining similar codes and grouping codes within broader categories (Saldaña, 2013; Miles et al., 2015). CB also developed an unconstrained coding matrix based on the conceptual framework of financial barriers to care for adults with chronic cardiovascular conditions (Campbell et al., 2016). This deductive coding matrix was incorporated into the inductively developed code structure, and the resulting codebook was applied to another 20% of interviews. Codes were developed for relevant sections of text that could not be categorized within the existing scheme, and the updated codebook was reviewed and refined by other research team members.

In second cycle coding, CB and AW used consensus coding to apply this coding scheme to all transcripts, including those used in first cycle coding. CB and AW first independently coded a single transcript and then compared code applications, reflected on unrecognized assumptions or interpretations, resolved disagreements, and updated the codebook as needed. Once consensus was achieved, CB applied the codebook to all remaining interviews, and AW reviewed code applications, noting additional codes that should be applied and points of disagreement, with the ultimate goal of ensuring critical thinking in the code application process. A third coder (RA) was consulted in the case that disagreements could not be resolved. The final codebook is included in Supplemental Appendix 2.

Finally, coded excerpts were interpreted in relation to the original research questions, identifying resonant themes across interviews. Though analysis took place throughout the coding process, the review of coded excerpts took place after all code assignments were finalized. Particular attention was given to “pressure points,” defined as positive or negative experiences that change how an individual navigates a system (Schaal et al., 2016; Black, 2022), in order to connect individual experiences to systemic factors. Transcripts were marked by participant characteristics (i.e., cancer type and year of diagnosis, age, race, ethnicity, annual income, marital status, health insurance status at diagnosis and currently) such that excerpts were viewed in the context of interviewees’ identities and life circumstances. Resulting themes were used to adapt an existing conceptual framework of patient experiences facing financial barriers to care (Campbell et al., 2016), which was then revised iteratively through discussions with the research team and patient advocates, including attendees of the 2022 Patient Insight Congress, a gathering of advocates, healthcare professionals, and researchers hosted by the Patient Advocate Foundation.

## 3. Results

We completed 20 audio-only interviews averaging 43 min (range: 28–60 min; intended interview length: 30–45 min). Of the 20 adults interviewed, 70% identified as women (25% men, 5% gender non-conforming); 55% had a college degree; and 55% were single, divorced, or separated. The majority of interviewees identified as non-Hispanic white (60%), followed by Black or African American (20%), Hispanic or Latino (10%), Asian (5%), and Native Hawaiian or other Pacific Islander (5%). When asked to report their annual household income, 20% reported making less than $24,000, 40% between $24,000 and $47,999, and 35% $48,000 or more (5% did not disclose). Interviewees were diagnosed with a range of cancer types (with breast cancer most common, 40%) between 2012 and 2020 (median time since diagnosis = 4.5 years). At the time of diagnosis, the majority of interviewees were privately insured (60%), and 15% were uninsured. At the time of the interview, Medicare was the primary insurer for 55% of participants, followed by private insurance (35%) and Medicaid (10%) (Table 1).

**Table 1:** Characteristics of interviewed adults with a history of cancer (*N* = 20).

Participant characteristics	*N* (%)
**Age**	
19–35	2 (10%)
36–55	9 (45%)
56–75	9 (45%)
**Race/ethnicity**	
Non-Hispanic White	12 (60%)
Black or African American	4 (20%)
Hispanic White	2 (10%)
Asian	1 (5%)
Native Hawaiian or other Pacific Islander	1 (5%)
**Gender**	
Woman	14 (70%)
Man	5 (25%)
Gender non-conforming	1 (5%)
**Sexual orientation**	
Heterosexual	17 (85%)
LGBTQIA+	2 (10%)
Prefer not to say	1 (5%)
**Education**	
HS, GED, Other	2 (10%)
Some college or 2-year degree	7 (35%)
College degree or more	11 (55%)
**Marital status**	
Single, divorced, or separated	11 (55%)
Married or partnered	9 (45%)
**Annual household income**	
Less than $24,000	4 (20%)
Between $24,000 and $47,999	8 (40%)
$48,000 or more	7 (35%)
Prefer not to say	1 (5%)
**Current employment status**	
Disabled, not able to work	9 (45%)
Retired or not employed	4 (20%)
Employed full-time by someone else	3 (15%)
Self-employed	3 (15%)
Employed part-time by someone else	1 (5%)
**Cancer type**	
Breast	8 (40%)
Multiple myeloma	3 (15%)
Blood	3 (10%)
Head and neck	2 (5%)
Other*	4 (20%)
**Time since diagnosis**	
1–2 years	7 (35%)
3–4 years	5 (25%)
5–6 years	5 (25%)
More than 6 years	3 (15%)
**Insurance at diagnosis**	
Private	12 (60%)
Medicare/medicaid/tricare	5 (25%)
Uninsured	3 (15%)
**Current insurance**	
Medicare	11 (55%)
Private	7 (35%)
Medicaid	2 (10%)

* Other includes colorectal, lung, ovarian, and gastrointestinal.

Cancer-related financial hardship led to the majority of interviewees either delaying or forgoing medical care, including diagnostic procedures (30%), primary cancer treatment (30%), supportive medications and therapies (50%), surveillance/monitoring (5%), and care for other conditions (30%) (Table 2). For example, one interviewee described forgoing supportive medications – “There were a couple meds that I could not afford to get… I just had to pick and choose…” (05: 56–75 years old, Stage 3 multiple myeloma). Direct causes of delayed and forgone care included out-of-pocket medical cost uncertainty, services or medications not being covered by insurance, prohibitive patient cost sharing (i.e., deductible, coinsurance, co-pays), and uninsurance. Non-medical cost barriers, such as not being able to take time off work or afford transportation to access care, were reported as challenges, but less commonly identified as causing participants to delay or forgo care.

**Table 2:** Types of cost-related delayed and forgone care described by interviewees with a history of cancer.

Type of care	Illustrative quotation
Diagnostic	“When I was initially diagnosed with cancer, it was due to a diagnostic mammogram, which I delayed because I did not have $300 to pay for it…and so while my cancer was caught, you know, in stage two, I do go back and think about, I should have just done that diagnostic mammogram.” (13: 36–55 years old, Stage 2 breast)
Cancer treatment	“I know what cancer is, and it just all hit me that I would not be able to follow through with any of it without money.” (12: 56–75 years old, Stage 3 multiple myeloma)
Supportive care	“There were a couple meds that I could not afford to get…I just had to pick and choose…I would sacrifice the pain med and another one until I could work out how I could afford to get that thing.” (05: 56–75 years old, Stage 3 multiple myeloma)
Surveillance	“So I’m actually due to have [an MRI] coming up in a few months, but I’m not going to have insurance anymore… it’s quite possible I’m not going to be able to get that this year, which really sucks because I definitely need it.” (09: 36–55 years old, Stage 3 breast)
Other medical care	“I went to CVS to get my [multiple sclerosis] prescription refilled and it was gonna cost $395. So I just refused. I said, well, I’ll just have to go without it.” (06: 56–75 years old, blood)

**Figure 1:**
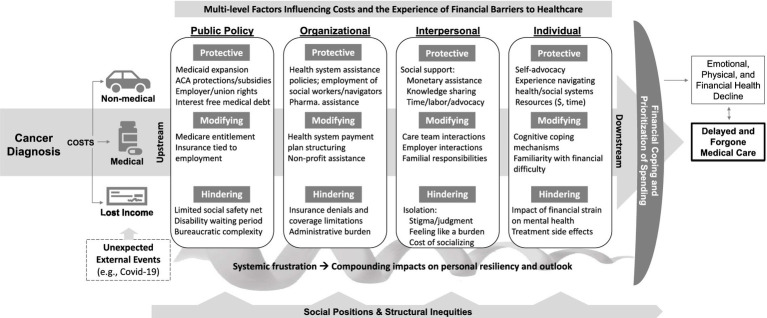
This framework displays for understanding the experience of financial barriers to healthcare by adults with a history of cancer. This framework displays the multi-level factors influencing patient experiences facing financial barriers to healthcare. Protective factors lessened the causes and consequences of financial barriers, hindering factors exacerbated them, and modifying factors had the potential to be either protective or hindering.

In addition to describing experiences delaying and forgoing care due to cost, interviewees described in-depth the causes of the financial barriers experienced, their process of coping with or attempting to overcome these barriers, and the consequences of this process, on both delayed and forgone care, as well as their physical, emotional, and financial health. Figure 1 organizes emergent themes into a conceptual model of the multi-level protective, modifying, and hindering factors influencing patients’ experiences facing financial barriers to medical care. Protective factors lessened the causes and consequences of financial barriers, hindering factors exacerbated them, and modifying factors had the potential to be either protective or hindering (Campbell et al., 2016). This model serves as an organizing framework for the factors we identified as influencing patient experiences of financial barriers to care in our analysis. Additionally, it presents an opportunity to stimulate future research on this topic. Below, we describe emergent qualitative themes, organized by model component.

### 3.1. Costs

Figure 1 depicts costs incurred as a consequence of cancer leading to financial barriers to care. In addition, factors influencing patient experiences facing financial barriers can also affect the magnitude of the costs incurred (e.g., the time required to find and navigate resources and medical care leading to more time off work). The amount and impact of cancer-related costs were also influenced by unexpected external events, most notably the Covid-19 pandemic. Interviewees described no longer being able to rely on childcare from family members and public transportation, thus increasing non-medical costs. The pandemic also caused several interviewees and their caregivers to lose employment, leading to insurance churn and lost income. *“When I lost my job [due to Covid-19], I felt the full effect of cancer and my job and just, everything just fell apart”* (17: 36–55 years old, Stage 4 breast).

### 3.2. Multi-level factors influencing costs and the experience of financial barriers to healthcare

#### 3.2.1. Public policy factors

Though interviews focused on participants’ individual experiences, the influence of policies in both protecting interviewees from financial burden and exacerbating it shone through, with particular emphasis on the shortcomings of governmental protections. Each key protection mentioned came with caveats. For example, though Medicaid provided comprehensive coverage for those able to qualify, participants expressed frustration over the strict eligibility criteria, particularly in non-expansion states [“*when I tried [to apply for Medicaid] first, my husband and I were not eligible because he was still working… [and made] like a hundred dollars more than the limit”* (12: 56–75 years old, Stage 3 multiple myeloma)]. Similarly, the Patient Protection and Affordable Care Act provided important protections for individuals with pre-existing conditions; however, threats of these protections being overturned still led one interviewee to reflect, *“I did not want to be diagnosed…because I was afraid I wasn’t going to get the pre-existing condition protection”* (02: 36–55 years old, Stage 4 breast). Other avenues of acquiring insurance, such as Medicare entitlement for individuals with qualifying disabilities and employer-sponsored insurance, also came with notable limitations, such as administrative delays, prohibitive out-of-pocket costs, and insurance churn. Additional factors at the public policy level are outlined in Table 3, along with illustrative quotations.

**Table 3:** Public policy factors influencing the experience of financial barriers to healthcare.

Factor	Illustrative quotation
*Protective*	
Medicaid expansion	“I did not really have any issues with, with Medicaid that, like I said, it was, everything was covered, you know?” (01: 56–75 years old, Stage 2 lung)
Affordable Care Act protections and subsidies	“I was really worried that you know I wasn’t gonna get covered, because I was going to graduate soon, so I could not be I did not want to be diagnosed or have it on the radar because I was afraid I wasn’t going to get the pre-existing condition protection.” (02: 36–55 years old, Stage 4 breast)
Employer/union rights	“Because I had been with the, the college for over 20 years, I was able to carry that health insurance with me and for my wife afterwards…it was a union contract from 15 years previous.” (15: 36–55 years old, Stage 3 multiple myeloma)
Interest-free medical debt	“There’s no interest on medical debt…. I knew how the laws worked with medical debt. You know because the hospital, they can be like really kind of bullies…” (04: 36–55 years old, Stage 4 colorectal)
*Modifying*	
Medicare entitlement	“And you know, with Medicare, the copay for part B is like 20%. That copay for my treatment was about $2,000 to $3,000 every 3 weeks. And if it got to the point where I’m trying to figure out how to pay for this.” (01: 56–75 years old, Stage 2 lung)
Insurance tied to employment	“So, when I lost my job like I said, that was not the time to lose my job because I’m, you know, I’m not married. So no health insurance. And it was like, Okay, this is how I’m gonna die. I’m gonna die of Covid because, you know, Covid wiped my job out.” (17: 36–55 years old, Stage 4 breast)
*Hindering*	
Limited social safety net	“The amount that [social security disability] gives you per month is not really a livable amount, and then they limit you on what you can earn per month.” (05: 56–75 years old, Stage 3 multiple myeloma)
Disability waiting period	“You have to wait 6 months to get any kind of disability payments and then 2 years for Medicare, which is pretty frustrating because at that point, you are in a bad situation… I just think they are hoping that people will die off or not need it anymore.” (04: 36–55 years old, Stage 4 colorectal)
Bureaucratic complexity	“And then I did the paperwork [for SNAP] because there’s supposed to be an exception for people with disabilities. But now with the Covid shutdown, you have to do everything online and the application is like 40 pages. When they looked at it, I tried to call them back and said, you did not read the part that says I’m disabled, which increases the income level, but they did not know about that. So I still have that appointment to visit in person.” (01: 56–75 years old, Stage 2 lung)

#### 3.2.2. Organizational factors

Organizational factors related to healthcare systems, insurance companies, pharmaceutical manufacturers, and non-profit organizations. Protective organizational factors included healthcare system financial assistance programs and the employment of a sufficient number of social workers and financial navigators to assist patients in understanding and coping with the cost of care. As described by one interviewee, “*the oncology unit has social workers attached to it…when you first have a cancer diagnosis they sort of flood you with resources*” (07: 56–75 years old, Stage 4 breast). Financial assistance programs through pharmaceutical manufacturers, and the connection to these resources through care team members, were also protective. The availability of payment plans through healthcare systems could be protective, warding off collections and medical debt, but several interviewees described instances in which they felt pressured by the healthcare system to pay more than they could afford each month – “*the hospital, they can be really kind of bullies…like you are going to pay this or we’ll send you to collections*” (04: 36–55 years old, Stage 4 colorectal). Non-profit assistance was similarly an important resource, but it was not guaranteed and often came with very specific eligibility criteria, complex applications, and funding limits [“*if they do not have the availability in your disease fund, then you are out of luck*” (20: 56–75 years old, blood)].

Insurance denials, processing delays, and coverage limitations were described as sources of substantial frustration by the majority of interviewees. This frustration was underscored by a sense of injustice that insurance coverage determinations ultimately determined care decisions instead of their oncologist or other care team members. Outstanding denied charges and concerns about future denials weighed heavily on interviewees, particularly in instances in which accessing treatment was a matter of survival. “*Whether the insurance company does not approve a new drug or a clinical trial or anything like that is terrifying to me… that’s always in the back of my mind*” (10: 19–35 years old, Stage 4 breast). Though caused by organizational practices and policies, the administrative burden of communicating between the fragmented landscape of organizations providing and paying for medical care was ultimately felt by patients and their caregivers [“*it wound up being a lot on me just going back and forth with different people in the insurance company*” (09: 36–55 years old, Stage 3 breast)]. An overview of organizational factors and illustrative quotations are included in Table 4.

**Table 4:** Organizational factors influencing the experience of financial barriers to healthcare.

Factor	Illustrative quotation
*Protective*	
Health system assistance policies	“… sometimes the hospitals, if you even just ask for assistance, will give you some somewhat of a reduced bill.” (04: 36–55 years old, Stage 4 colorectal)
Employment of social workers and navigators	“The oncology unit has social workers attached to it…when you go into [health system] and you first have a cancer diagnosis, they sort of flood you with resources.” (07: 56–75 years old, Stage 4 breast)
Pharmaceutical assistance	“I need the med to stay alive, but it’s so expensive that you cannot afford it without a grant or, or something. You know, this ain’t gonna work. So I guess at this point, it, it has worked itself out. But yeah, about every 10, 11 months I have to worry about it.” (05: 56–75 years old, Stage 3 multiple myeloma)
*Modifying*	
Health system payment plan structuring	“I chose to go on a payment plan and I pay $5 a month… I used to see [my hematologist] every couple of months, but she said she did not need to see me for 6 months. And I’m wondering if that has anything to do with…I do not have any clue if she knew that I had put myself on the $5 a month payment plan.” (06: 56–75 years old, blood)
Non-profit assistance	“Nonprofits. Yeah. Those really have been what have kept us afloat. It’s sad to say that it’s not been, you know, our, our government or any kind of, you know system. It’s been nonprofits that have really been there.” (04: 36–55 years old, Stage 4 colorectal)
*Hindering*	
Insurance denials and coverage limitations	“It’s so crazy how one insurance will say, you can take this medicine and then another insurance will say, nope, you gotta take this medicine. And to me, that’s like, why do not you just listen to what my doctor wants me to take?” (09: 36–55 years old, Stage 3 breast)
Administrative burden	“I’ve had to fight [with insurance] to get some medicines that help me get through the day.” (03: 19–35 years old, Stage 4 breast)

#### 3.2.3. Interpersonal factors

Interactions with care team members and employers had the potential to be either protective or hindering, depending on their quality. Positive interactions were characterized by interviewees feeling as though their financial concerns were seen and understood. Additionally, both care team members and employers had the potential to use the power associated with their positions in support of the patient. For example, interviewees described care team members advocating to insurance and pharmaceutical companies [“*my oncologist was pretty savvy – she was able to go to manufacturers and get different chemotherapies for me”* (14: 36–55 years old, Stage 3 ovarian)] and initiating disability applications on their behalf. They also described employers allowing scheduling flexibility around medical appointments. Familial responsibilities, particularly related to providing for children, could also be either protective or hindering, serving as a motivator or source of stress. “*I have to try to keep everything good for my family”* (04: 36–55 years old, Stage 4 colorectal).

Interviewees referenced the benefit of social support in protecting them from the full weight of financial barriers through direct monetary assistance [*“we did not go without because of our family”* (16: 36–55 years old, Grade 4 head and neck)]; knowledge sharing about available resources [*“a lot of my information I got thankfully from my support groups on Facebook”* (10: 19–35 years old, Stage 4 Breast)]; and time, labor, and advocacy [*“my mom helped me start the [disability] application process”* (13: 36–55 years old, Stage 2 breast)]. In contrast to social support, isolation – whether due to perceived stigma, not wanting to place burden on others, or not being able to afford socializing – worsened the experience of financial barriers. An overview of interpersonal factors and illustrative quotations are included in Table 5.

**Table 5:** Interpersonal factors influencing the experience of financial barriers to healthcare.

Factor	Illustrative quotation
*Protective*	
Social support: Monetary assistance	“We did not go without because of our family.” (16: 36–55 years old, Grade 4 head and neck)
Social support: Knowledge sharing	“A lot of my information I got thankfully from my support groups on Facebook.” (10: 19–35 years old, Stage 4 breast)
Social support: Time, labor, and advocacy	“I’ve had a lot of advocates fight for me, my husband, my mother, my mother-in-law.” (03: 19–35 years old, Stage 4 breast)
*Modifying*	
Care team interactions	“I have gone through a couple navigators. The one that really helped me…the key thing that made her so great…was that she was a social worker…. And then she left and I’m left with a new navigator, and she does not know anything…. I feel really lost without my old navigator right now.” (02: 36–55 years old, Stage 4 breast)
Employer interactions	“I had been there so long, and was such a good employee, that [supervisor] worked with me. I was able to do my chemo on a Friday, so I had all weekend to recover and then I was back Monday. And if my duties needed to be light, he would work with me.” (17: 36–55 years old, Stage 4 breast)
Familial responsibilities	“And I’m a single mom. I have a daughter, and so I need to make sure that she’s fed and taken care of.” (09: 36–55 years old, Stage 3 Breast)
*Hindering*	
Isolation: Stigma/judgment	“And the one thing I’m concerned about is like the stigma of lung cancer…. I cannot tell my family because they have been telling me that I need to stop smoking for years.” (01: 56–75 years old, Stage 2 lung)
Isolation: Feeling like a burden	“And you know, I just try to make sure that I do not have to inconvenience someone to the extent where financially they gotta be a, you know, it’s gonna be a burden on them as well.” (19: 56–75 years old, Stage 1 breast)
Isolation: Cost of socializing	“Everybody’s like, let us just go out to lunch. And I’m like, if I could afford to go out to lunch, I would be there, but I just, I cannot… I was going to the ovarian cancer support group. The only person in the financial boat that I’m in is me. Everybody else has a lot of money. So I actually quit that group because I just, I wasn’t feeling comfortable.” (14: 36–55 years old, Stage 3 ovarian)

#### 3.2.4. Individual factors

The most notable protective factor at the individual level was the ability to advocate on behalf of one’s own financial concerns to care team members, the health system billing office, insurance companies, and government social assistance programs. Underlying this tendency toward self-advocacy was a comfort asking for help (which interviewees described as requiring *“laying down pride”* (08: 36–55 years old, blood) and adjusting to a loss of independence). Additionally, self-advocacy required a baseline resource and health insurance literacy, facilitated for several interviewees by prior work in case management, insurance, or a healthcare system. Familiarity with financial difficulty and navigating social assistance systems in the past also facilitated this baseline knowledge. Furthermore, advocating for oneself required time, with several interviewees describing the process of searching for and applying for resources as akin to a full-time job. “*I cannot imagine how many people have given up because they did not have the time or energy to [navigate resources]*” (18: 56–75 years old, Stage 1 head and neck).

Interviewees described varying cognitive approaches to coping with the experience of financial barriers, ranging from avoidance and resignation [“*I wanted it to all go away*” (02: 36–55 years old, Stage 4 breast)] to being proactive and facing problems head on [“*the brain fog is clearing up and now I can be more active in finding solutions to my problems”* (01: 56–75 years old, Stage 2 lung)]. An underlying belief that resources were available facilitated problem-focused coping, whereas feelings of despair and overwhelm led to avoidance and resignation.

Physical, mental and emotional health challenges were described as limiting one’s capacity to overcome financial barriers to care. Physical and mental side effects of the cancer and treatment, most notably fatigue and “brain fog,” made the process of finding resources and completing complex applications more difficult. “*You’re fighting cancer, you just had pneumonia, do you really want to fight with insurance companies on the phone?*” (15: 36–55 years old, Stage 3 multiple myeloma). Further, financial strain often led to or exacerbated mental health challenges, which in turn limited one’s ability to overcome financial barriers. In addition to material financial burden, emotional health challenges stemmed from feeling discouraged and alone in the process of applying for resources, the psychological effect of seeing the cost of treatment needed to survive, reliance on an unreliable system, and concerns about the future (e.g., passing debt onto family members, the potential of treatment not being covered). An overview of factors operating at the individual level are included in Table 6, along with illustrative quotations.

**Table 6:** Individual factors influencing the experience of financial barriers to healthcare.

Factor	Illustrative quotation
*Protective*	
Self-advocacy	“Nobody else is gonna help you unless you do it yourself, so you contact as many people as you can. You exhaust every avenue and you research every possibility.” (18: 56–75 years old, Stage 1 head and neck)
Experience navigating health/social systems	“My background is in insurance…so I know the system somewhat and I can kind of talk the language at times. And I’m not afraid to do that.” (20: 56–75 years old, blood)
Resources (money, time)	“I did nothing but, every single day and night, I did nothing but research on the computer” (18: 56–75 years old, Stage 1 head and neck)
*Modifying*	
Cognitive coping mechanisms: Problem-focused	“When I get depressed or sad, I say, [name], take an hour and be depressed. Just take a whole hour. Who a is me, cry. Whatever you need to do, holler, scream. After that hour, go into action.” (20: 56–75 years old, blood)
Cognitive coping mechanisms: Avoidance, resignation	“Just the weight of it all made me want to sleep. Just sleep. I wanted it to all go away.” (02: 36–55 years old, Stage 4 breast)
Familiarity with financial difficulty	“So I had to learn to play with my money in ways that I never before had to.” (17: 36–55 years old, Stage 4 breast)
*Hindering*	
Impact of financial strain on mental health	“Mental health and financial health, they go together. You gotta have the funds in order to relax, not to be stressed. All the stress over the years… And I’m sure that not only with my disease but with other diseases, it leads to other diseases when you are financially stressed.” (20: 56–75 years old, blood)
Treatment side effects	“At that point, from the brain fog, I wasn’t in a position to really think clearly or to navigate the system all by myself.” (01: 56–75 years old, Stage 2 lung)

### 3.3. Systemic frustration

The hindering policy and organizational factors described above, paired with the precarity of existing protections, led many interviewees to express frustration toward governmental policies, insurance companies, and health systems. One interviewee described the complexity of the healthcare system as being *“designed to where you’ll give up”* and *“a comedy of errors designed not to pay”* (18: 56–75 years old, Stage 1 head and neck), perceiving an intentionality motivating the financial barriers they experienced. This frustration toward the systems and policies influencing cancer care costs had the potential to affect individual resiliency and outlook. For some interviewees, systemic frustration led to individual despair; in contrast, other interviewees reflected substantial frustration at the systemic level but maintained an individual determination fueled by motivation to survive and be around for family members. The extent to which interviewees felt that their financial challenges were seen and valued – whether by care team members, non-profit organizations, or friends and family – influenced the extent to which systemic frustration led to despair at an individual level. Additionally, having success finding resources, even if they were limited in nature, reinforced interviewees’ determination and belief that they could overcome the financial barriers experienced. *“All I needed was just a little bit of help to buy me time to get my stuff together because I’m a fighter, I’m gonna figure it out”* (17: 36–55 years old, Stage 4 breast).

### 3.4. Financial coping and prioritization of spending

In the face of resource constraints, interviewees described a somewhat constant process of having to prioritize how to allocate money, whether between medical care and household necessities [*“I had to choose between putting gas in the car or getting the medications”* (08: 36–55 years old, blood)], different types of medical care (e.g., primary treatment versus supportive therapies or medications), or spending for oneself or one’s children [*“I would sacrifice anything of mine before my kids would want for something”* (05: 56–75 years old, Stage 3 multiple myeloma)]. Though this process happened at the individual level, it resulted from the cumulative impact of multi-level factors and was shaped by interviewees’ resiliency and outlook.

Interviewees, particularly those who had not experienced financial difficulty in the past, described the impact of this prioritization process on their mental health. *“Sometimes I get very anxious because of not knowing which [necessity] you are gonna take care of…”* (06: 56–75 years old, blood). Others pointed out that forgoing household necessities, such as spending less on groceries or not paying for needed car repairs, instead of medical care also had an impact on their health and ability to get to their appointments. *“If I do not pay my car note…I need that to get to and fro because at one time my car was about to break down and I’m thinking, how am I gonna even get to treatment?”* (19: 56–75 years old, Stage 1 breast).

### 3.5. Emotional, physical, and financial health decline

Though the primary focus of our interviews was to understand patient experiences leading up to, or preventing, cost-related delayed and forgone medical care, it was clear that the process of coping with financial barriers to care, whether resulting in changes to healthcare utilization or not, had deleterious impacts on interviewees’ financial health (e.g., depleted savings, consolidation of debt, giving up on buying a house), as well as their physical and emotional health. Even among interviewees who prioritized medical care above all else, cutting back on grocery spending or relying on food banks had the potential to lead to a less nutritious diet, inability to afford a gym membership limited opportunities to exercise, and the emotional stress of seeking resources and the prioritization process described above resulted in physical consequences. For example, one participant reported a new hypertension diagnosis, stating, *“I was diagnosed with high blood pressure…all the stress over the years…it leads to other diseases when you are financially stressed”* (20: 56–75 years old, blood).

### 3.6. Social positions and structural inequities

Social positions – related to an individual’s socioeconomic status, age, race, ethnicity, and sexual and gender identity (among other factors) – influenced interviewees’ experiences of each of the multi-level factors described above. Interviewees’ positions, including intersecting positions along multiple dimensions of identity, had the potential to be associated with marginalization, advantage, and opportunities for strength and resilience (Fredriksen-Goldsen et al., 2014). Additionally, and related to social positionality, structural inequities – such as racism, discrimination, and social exclusion – create an inequitable distribution of power and resources, which shaped interviewees’ experiences with financial challenges and the ability to overcome them (Alcaraz et al., 2020). Examples described by interviewees included shame associated with using social services [*“I never thought I would come to a day where I would have to apply for the food stamp program…to me that’s somewhat embarrassing”* (20: 56–75 years old, blood)]; health system prioritization of patients with higher paying insurance [*“I was concerned, because of the fact that I did not have insurance, that I would not receive the proper care”* (08: 36–55 years old, blood)]; and discrimination from healthcare providers [*“I’m overweight, and I felt a little prejudiced… she [surgeon] made me feel like I did not deserve to get the procedure”* (02: 36–55 years old, Stage 4 breast)]. Social positions and structural inequities are included as underlying each of the other model components, as they influence each, with the potential to influence the extent to which financial barriers translate to deleterious physical, mental, and emotional health outcomes.

## 4. Discussion

Our findings describe the experiences of adults with a history of cancer coping with financial barriers to medical care, including their perspectives on the multi-level protective, hindering, and modifying factors influencing those experiences. Additionally, they demonstrate how the process of facing financial barriers to care influences patient physical and emotional health, both through cost-related delayed and forgone care, as well as independent of it. The adapted conceptual framework presented in Figure 1 is intended to inform multi-level intervention to lessen the financial barriers experienced and support patients in navigating health and social care systems to overcome them.

### 4.1. Qualitative findings

A key finding from our analysis was the influence of attempting to overcome financial barriers on individual emotional well-being. In particular, we describe the systemic frustration resulting from bureaucratic complexity of health and social care systems and resulting administrative burden. A qualitative study conducted among cancer survivors in Germany also identified the substantial influence of navigating a bureaucratic system, having insufficient resources and needing to ask for help on patient distress (Lueckmann et al., 2022). Frustration toward insurance companies dictating care decisions has also been documented among adults with cancer (Thomson and Siminoff, 2015), as well as the “logistic toxicity” of constantly searching for the lowest cost pharmacies for supportive medications (Etteldorf et al., 2022). Furthermore, several studies have highlighted the role of health insurance literacy – defined as the ability to obtain, understand and use health plan information (Paez et al., 2014) – in influencing overall patient financial hardship (Zhao et al., 2019; Khera et al., 2022) and delayed care in the absence of clear cost expectations (Waters et al., 2022).

In addition to the negative impact of coping with financial barriers to care on mental and emotional well-being, we found that the consequences of financial barriers – whether forgone care or other lifestyle changes – also had the potential to negatively impact mental and emotional well-being. This is in line with qualitative work conducted among cancer survivors in rural Australia, which described the potential negative impact of cost-saving strategies on individual enjoyment, access to social support, and well-being (Skrabal Ross et al., 2021). In turn, our findings also illustrate the role of mental health and emotional well-being in either supporting or hindering individuals in attempting to overcome financial barriers experienced, creating a feedback loop. This relationship is supported by an analysis of cancer survivors surveyed in the Cancer Survivorship Supplement of the Medical Expenditure Panel Survey, which found that patient-reported financial worry attenuated the association between financial difficulty and positive coping behaviors, suggesting that participants with high financial worry were less likely to use positive coping strategies to mitigate financial difficulties (Jones et al., 2018).

### 4.2. Adapted conceptual framework

Though our study was influenced by a conceptual framework of financial barriers to healthcare developed among adults with chronic cardiovascular conditions, our adapted framework diverges in several key ways based on our qualitative findings. First, we delineate multi-level influences in line with a socio-ecological framework. This allows us to frame individual outlook and resiliency – important components of both models – as being influenced by systemic factors rather than operating solely at the level of the individual. We also introduce the concept of prioritization, which involves determining how to allocate limited resources between different types of medical care and medical versus non-medical needs (e.g., mortgage, car payments). As a result, our model also includes non-clinical consequences of facing financial barriers to care. This is in line with prior work documenting high willingness to sacrifice both personally and financially for cancer care, especially among patients with metastatic disease (Chino et al., 2018).

In contrast to the model developed by Campbell and colleagues, which included mental illness and physical limitations as “predisposing” factors, we found that mental health challenges and physical limitations described by interviewees in our study were largely consequences of the cancer diagnosis, treatment, and associated financial hardship. As such we renamed these as hindering factors to include both factors caused by a cancer diagnosis and associated costs, as well as underlying mental and physical comorbidities that may exacerbate financial hardship experienced. This is in line with the broader psycho-oncology literature, which has documented both the consequences of cancer-related financial hardship on mental health (Inguva et al., 2022; Pangestu and Rencz, 2023), as well as the influence of underlying mental health comorbidities on cancer care access and outcomes (Baillargeon et al., 2011; Giese-Davis et al., 2011; Rieke et al., 2017). We also found that familiarity with financial difficulties, categorized as protective by Campbell and colleagues, could be either protective or hindering, depending on whether the interviewee was experiencing financial difficulty at the time of diagnosis.

Our interpretation of findings was also informed by existing conceptual frameworks of financial burden developed in the cancer context, with a particular focus on Jones and colleagues’ theoretical model of financial burden after cancer diagnosis (Jones et al., 2020). As conceptualized by this model, our analysis studied the pathway between causes of financial burden and healthcare-specific financial coping behaviors (i.e., cost-related care interference). Based on patient experiences facing financial barriers to care analyzed in our study, we broadened the conceptualization of cost-related care interference to include prioritization, in addition to coping. This highlights the interrelated nature of approaches to reduce medical and non-medical costs and the inherent tradeoffs patients must face.

### 4.3. Implications

Our findings and the adapted conceptual framework present opportunities for intervention to both reduce the costs incurred, and thus financial barriers faced, as well as to support patients in navigating financial barriers experienced. The further upstream, or more systemic, the intervention, the more likely it will be to reduce current barriers preventing equitable access to cancer care. Examples of policy and regulatory changes that could substantially reduce the financial barriers to care experienced by adults with cancer in the United States include Medicaid expansion in states that have not yet, policies promoting containment of medical and pharmaceutical costs [e.g., Inflation Reduction Act (Shih et al., 2023)], and enforcement of community benefit obligations of not-for-profit hospitals (Patient Protection and Affordable Care Act, 2010; Doherty et al., 2022). Additionally, upholding and building upon patient protections passed with the 2010 Affordable Care Act is critical, particularly for cancer survivors, 190,000 of whom lost health insurance due to the erosion of such protections following the 2016 United States elections (Moss et al., 2020). Though we focus on United States policy implications, as our study was conducted among patients navigating the United States healthcare system, it is important to note that financial barriers to healthcare, particularly those related to the non-medical costs associated with a cancer diagnosis and associated care, are experienced by adults with cancer across the world, even in countries with universal healthcare coverage (Barbaret et al., 2017; Garaszczuk et al., 2022). Policies related to employment protections and social income support may be particularly important to reducing financial hardship experienced in these contexts (Sayani et al., 2021).

At the organizational level, health systems must make hospital-based financial assistance more accessible and eligibility criteria more transparent to promote equitable access to available resources. A recent qualitative brief described patient barriers to accessing financial assistance, including “lack of awareness, perceptions of ineligibility, fear of negative consequences, and being overwhelmed” (Doherty et al., 2022). Interviewees in our analysis described similar sentiments. Additionally, implementation of robust oncology financial navigation programs, proactively offered to all patients receiving cancer care, has the potential to systematically lift administrative burden and advocacy responsibilities off of patients and caregivers. Preliminary evidence has shown that financial navigation reduces patient financial hardship, (Wheeler et al., 2020; Doherty et al., 2021) improves patient satisfaction (Doherty et al., 2021), and may also improve health system revenue (Yezefski et al., 2018).

### 4.4. Strengths and limitations

This study must be viewed in the context of several strengths and limitations. First, we employed a template analysis within a pragmatic paradigm, involving several stages of analysis. Given that template analysis is a flexible approach that does not have an inherent philosophical position, it is possible that it could lead to superficial findings if used by inexperienced qualitative researchers with little knowledge of cancer-related financial hardship. However, our multi-disciplinary team included several experienced qualitative researchers and substantial expertise in various aspects of financial hardship. In turn, the flexibility of template analysis allowed us to employ a variety of qualitative paradigms to both align findings with participant lived experiences while also situating them in the context of existing literature. Another limitation of the methodology employed is that we did not engage in member checking or reflections (i.e., providing qualitative findings back to participants for feedback and corrections; Tracy, 2010) which limits our certainty in the interpretation of participant experiences. However the rigor and reliability of the multi-stage qualitative analysis, involving discussing findings and interpretation with patient advocates, lends credibility to our findings. Finally, our presentation of qualitative findings and an adapted conceptual framework together allows for readers to gain a better understanding of how the framework was conceptualized and examples of constructs via participants quotations.

Interviewees identified through PAF may not be representative of cancer survivors as a whole, given that they had already accessed at least one external resource, whether on their own or through a care team member. Furthermore, individuals willing and able to participate in an interview about their experiences may be mentally and physically healthier than the broader population of adults with a history of cancer. As a result, our findings may not reflect the full extent of the relationship between cancer-related financial hardship, financial barriers to healthcare, and mental illness. Future research should focus on this association specifically, particularly in light of concerning data showing an association between financial strain and suicide attempts (Elbogen et al., 2020).

Additionally, reaching participants by email may also bias the sample toward those that are technologically literate. However, given that the purpose of our study was to understand the experience of facing, and in some cases overcoming, financial barriers to care, this sample was well-suited to our research question. It is possible that recall bias influenced interviewee responses, given that the median time since diagnosis was 4.5 years, but a substantial portion of the interviews focused on interviewee’s current experiences and those in the prior year, as the screener questions assessing financial hardship were based on the prior year. Lastly, our study focused on the patient perspective, but this is not meant to ignore the role of caregivers in navigating financial barriers to care with, or on behalf of, patients. Future research should apply and adapt this conceptual framework in the caregiver context, particularly given recent findings documenting spillover cost-related delayed and forgone care among family members of patients with cancer (Kazzi et al., 2022).

## 5. Conclusion

Despite individual motivation, knowledge, and support to access resources, interviewees facing financial barriers were limited by a constrained resource context characterized by impermanence, delays, administrative hurdles, and strict eligibility criteria. This study adapts the only existing conceptual framework of financial barriers to care to adult cancer survivors. Though our conceptual framework is not meant to be exhaustive or final, it presents an important opportunity for future research building on our understanding and conceptualization of patient experiences as they cope with cancer care costs and attempt to overcome financial barriers to needed medical care. It also serves as a useful framework for mapping multi-level interventions designed to reduce patient financial hardship and, ultimately, deleterious, inequitable health outcomes. Specifically, the framework points to the importance of upstream (policy and organizational) interventions, such as cost containment policies and systematic financial navigation programs, to reduce the administrative and financial burden experienced by patients and their caregivers.

## Data availability statement

The original contributions presented in the study are included in the article/Supplementary material, further inquiries can be directed to the corresponding author.

## Ethics statement

The studies involving human participants were reviewed and approved by University of North Carolina at Chapel Hill Institutional Review Board (UNC-CH IRB#22-0467). Written informed consent for participation was not required for this study in accordance with the national legislation and the institutional requirements.

## Author contributions

CB, RA, KG, EK, LS, DR, AP, and SW contributed to the conception and design of the study. CB, RA, and KG collected the data. CB and AW conducted the qualitative analysis. CB wrote the first draft of the manuscript. All authors contributed to the article and approved the submitted version.

## Funding

CB and AW were supported by a Cancer Care Quality Predoctoral Traineeship, National Cancer Institute (NCI), grant no. T32-CA-116339, for which SW is mentor and PI. CB was also supported by the Emotional Well-being and Economic Burden (EMOT-ECON) Research Network Dissertation Research Award. The EMOT-ECON Research Network is funded by a grant awarded by the National Center for Complementary and Integrative Health (NCCIH), the Office of Behavior and Social Sciences Research (OBSSR), the Office of Disease Prevention and National Institutes of Health Office of the Director (U24AT011310-01).

## Conflict of interest

SW and DR have received research grants from Pfizer paid to their institution for unrelated work. LS and SW have received salary support from AstraZeneca paid to their institution for unrelated work.

The remaining authors declare that the research was conducted in the absence of any commercial or financial relationships that could be construed as a potential conflict of interest.

## Publisher’s note

All claims expressed in this article are solely those of the authors and do not necessarily represent those of their affiliated organizations, or those of the publisher, the editors and the reviewers. Any product that may be evaluated in this article, or claim that may be made by its manufacturer, is not guaranteed or endorsed by the publisher.

## References

[ref1] al Rowas S. Rothberg M. B. Johnson B. Miller J. AlMahmoud M. Friderici J.. (2017). The association between insurance type and cost-related delay in care: a survey. Am. J. Manag. Care 23, 435–442. PMID: 28817783 PMC5875437

[ref2] Alcaraz K. I. Wiedt T. L. Daniels E. C. Yabroff K. R. Guerra C. E. Wender R. C. (2020). Understanding and addressing social determinants to advance cancer health equity in the United States: a blueprint for practice, research, and policy. CA Cancer J. Clin. 70, 31–46. doi: 10.3322/caac.21586, PMID: 31661164

[ref3] Altice C. K. Banegas M. P. Tucker-Seeley R. D. Yabroff K. R. (2017). Financial hardships experienced by cancer survivors: a systematic review. J. Natl. Cancer Inst. 109:djw205. doi: 10.1093/jnci/djw205 27754926 PMC6075571

[ref4] Amin K Claxton G Ramirez G Cox C. How Does Cost Affect Access to Care? Kaiser Family Foundation. Health System Tracker. (2021). Available at: https://www.healthsystemtracker.org/chart-collection/cost-affect-access-care/#item-start. (Accessed September 15, 2021).

[ref5] Amir Z. Wilson K. Hennings J. Young A. (2012). The meaning of cancer: implications for family finances and consequent impact on lifestyle, activities, roles and relationships. Psychooncology 21, 1167–1174. doi: 10.1002/pon.2021, PMID: 21769990

[ref6] Archer M Bhaskar R Collier A Lawson T Norrie A. Critical Realism: Essential Readings. London: Routledge; (1998).

[ref7] Baillargeon J. Kuo Y.-F. Lin Y.-L. Raji M. A. Singh A. Goodwin J. S. (2011). Effect of mental disorders on diagnosis, treatment, and survival of older adults with Colon Cancer. J. Am. Geriatr. Soc. 59, 1268–1273. doi: 10.1111/j.1532-5415.2011.03481.x, PMID: 21732924 PMC4006964

[ref8] Barbaret C. Brosse C. Rhondali W. Ruer M. Monsarrat L. Michaud P.. (2017). Financial distress in patients with advanced cancer. PLoS One 12:e0176470. doi: 10.1371/journal.pone.0176470, PMID: 28545063 PMC5436643

[ref9] Black K. Z. (2022). “Equity-centered approaches to qualitative research” in Qualitative Research Summer Intensive. ed. ResearchTalk, Inc.

[ref10] Campbell D. J. Manns B. J. Leblanc P. Hemmelgarn B. R. Sanmartin C. King-Shier K. (2016). Finding resiliency in the face of financial barriers: development of a conceptual framework for people with cardiovascular-related chronic disease. Medicine (Baltimore) 95:e5561. doi: 10.1097/MD.0000000000005561, PMID: 27930562 PMC5266034

[ref11] Chino F. Peppercorn J. M. Rushing C. Nicolla J. Kamal A. H. Altomare I.. (2018). Going for broke: a longitudinal study of patient-reported financial sacrifice in Cancer care. J. Oncol. Pract. 14, e533–e546. doi: 10.1200/JOP.18.00112, PMID: 30138052 PMC6550053

[ref12] Clark A. M. MacIntyre P. D. Cruickshank J. (2007). A critical realist approach to understanding and evaluating heart health programmes. Health (London) 11, 513–539. doi: 10.1177/1363459307080876 17855471

[ref13] Dedoose. Dedoose Version 9.0.62, Web Application for Managing, Analyzing, and Presenting Qualitative and Mixed Method Research Data [Computer Program]. Los Angeles, CA: SocioCultural Research Consultants, LLC; (2022).

[ref14] Doherty M. Jacoby J. Gany F. (2022). “I wish I knew about these programs before!” a brief report exploring barriers to financial assistance reported by gynecological oncology patients. J. Psychosoc. Oncol. 14, 1–9. doi: 10.1080/07347332.2022.2149374 [Epub ahead of print]. PMC10322634 36514954

[ref15] Doherty M. J. Thom B. Gany F. (2021). Evidence of the feasibility and preliminary efficacy of oncology financial navigation: a scoping review. Cancer Epidemiol. Biomark. Prev. 30, 1778–1784. doi: 10.1158/1055-9965.EPI-20-1853 PMC9022465 34341051

[ref16] Elbogen E. B. Lanier M. Montgomery A. E. Strickland S. Wagner H. R. Tsai J. (2020). Financial strain and suicide attempts in a nationally representative sample of US adults. Am. J. Epidemiol. 189, 1266–1274. doi: 10.1093/aje/kwaa146, PMID: 32696055

[ref17] Etteldorf A. Sedhom R. Rotolo S. M. Vogel R. I. Booth C. M. Blaes A. H.. (2022). The least costly pharmacy for cancer supportive care medications over time: the logistic toxicity of playing catch up. Support Care Cancer 31:3. doi: 10.1007/s00520-022-07472-x, PMID: 36512134 PMC9745713

[ref18] Fredriksen-Goldsen K. I. Simoni J. M. Kim H. J. Lehavot K. Walters K. L. Yang J.. (2014). The health equity promotion model: reconceptualization of lesbian, gay, bisexual, and transgender (LGBT) health disparities. Am. J. Orthopsychiatry 84, 653–663. doi: 10.1037/ort0000030, PMID: 25545433 PMC4350932

[ref19] Garaszczuk R. Yong J. H. E. Sun Z. de Oliveira C. (2022). The economic burden of Cancer in Canada from a societal perspective. Curr. Oncol. 29, 2735–2748. doi: 10.3390/curroncol29040223, PMID: 35448197 PMC9025082

[ref20] Giese-Davis J. Collie K. Rancourt K. M. Neri E. Kraemer H. C. Spiegel D. (2011). Decrease in depression symptoms is associated with longer survival in patients with metastatic breast cancer: a secondary analysis. J. Clin. Oncol. 29, 413–420. doi: 10.1200/JCO.2010.28.4455, PMID: 21149651 PMC3058287

[ref21] Inguva S. Priyadarshini M. Shah R. Bhattacharya K. (2022). Financial toxicity and its impact on health outcomes and caregiver burden among adult cancer survivors in the USA. Future Oncol. 18, 1569–1581. doi: 10.2217/fon-2021-1282, PMID: 35129377

[ref22] Jiang H. Lyu J. Mou W. Jiang L. Zeng Y. Liu Y.. (2022). Prevalence and risk factors of self-reported financial toxicity in cancer survivors: a systematic review and meta-analyses. J. Psychosoc. Oncol. 1-18, 1–18. doi: 10.1080/07347332.2022.2142877 36370039

[ref23] Jones S. M. Henrikson N. B. Panattoni L. Syrjala K. L. Shankaran V. (2020). A theoretical model of financial burden after cancer diagnosis. Future Oncol. 16, 3095–3105. doi: 10.2217/fon-2020-0547, PMID: 32976048 PMC7787147

[ref24] Jones S. M. W. Walker R. Fujii M. Nekhlyudov L. Rabin B. A. Chubak J. (2018). Financial difficulty, worry about affording care, and benefit finding in long-term survivors of cancer. Psychooncology 27, 1320–1326. doi: 10.1002/pon.4677, PMID: 29462511 PMC13088915

[ref25] Kazzi B. Chino F. Kazzi B. Jain B. Tian S. Paguio J. A.. (2022). Shared burden: the association between cancer diagnosis, financial toxicity, and healthcare cost-related coping mechanisms by family members of non-elderly patients in the USA. Support Care Cancer 30, 8905–8917. doi: 10.1007/s00520-022-07234-9, PMID: 35877007

[ref26] Khera N. Zhang N. Hilal T. Durani U. Lee M. Padman R.. (2022). Association of Health Insurance Literacy with Financial Hardship in patients with Cancer. JAMA Netw. Open 5:e2223141. doi: 10.1001/jamanetworkopen.2022.23141, PMID: 35877122 PMC9315419

[ref27] King N Brooks JM. Template Analysis for Business and Management Students. In: 55 City Road, London; (2017). Available at: https://methods.sagepub.com/book/template-analysis-for-business-and-management-students. (Accessed March 31, 2023).

[ref28] King N Brooks J Cassell C Cunliffe A Grandy G. The SAGE Handbook of Qualitative Business and Management Research Methods: Methods and Challenges. Chapter 14: Thematic Analysis in Organisational Research. London, UK: SAGE Publications, Inc; (2018).

[ref29] Knight T. G. Deal A. M. Dusetzina S. B. Muss H. B. Choi S. K. Bensen J. T.. (2018). Financial toxicity in adults with Cancer: adverse outcomes and noncompliance. J. Oncol. Pract. 14, e665–e673. doi: 10.1200/JOP.18.00120, PMID: 30355027

[ref30] Levesque J.-F. Harris M. F. Russell G. (2013). Patient-centred access to health care: conceptualising access at the interface of health systems and populations. Int. J. Equity Health 12:18. doi: 10.1186/1475-9276-12-18 23496984 PMC3610159

[ref31] Lueckmann S. L. Schumann N. Kowalski C. Richter M. (2022). Identifying missing links in the conceptualization of financial toxicity: a qualitative study. Support Care Cancer 30, 2273–2282. doi: 10.1007/s00520-021-06643-6, PMID: 34716793 PMC8795015

[ref32] Maldonado J. A. Fu S. Chen Y. S. Acquati C. Yabroff K. R. Banegas M. P.. (2021). Sensitivity of psychosocial distress screening to identify Cancer patients at risk for financial hardship during care delivery. JCO Oncol Pract. 17, e1856–e1865. doi: 10.1200/OP.20.01009, PMID: 34043452 PMC8678032

[ref33] Malterud K. Siersma V. D. Guassora A. D. (2016). Sample size in qualitative interview studies: guided by information power. Qual. Health Res. 26, 1753–1760. doi: 10.1177/1049732315617444 26613970

[ref34] Miles MB Huberman AM Saldaña J. Qualitative Data Analysis: A Methods Sourcebook, 3rd. Thousand Oaks, CA: SAGE Publications, Inc.; (2015).

[ref35] Moon K. Blackman D. (2014). A guide to understanding social science research for natural scientists. Conserv. Biol. 28, 1167–1177. doi: 10.1111/cobi.12326, PMID: 24962114

[ref36] Moss H. A. Han X. Yabroff K. R. Chino J. Chino F. (2020). Declines in health insurance among cancer survivors since the 2016 US elections. Lancet Oncol. 21:e517. doi: 10.1016/S1470-2045(20)30623-9, PMID: 33069279 PMC8012006

[ref37] Paez K. A. Mallery C. J. Noel H. Pugliese C. McSorley V. E. Lucado J. L.. (2014). Development of the health insurance literacy measure (HILM): conceptualizing and measuring consumer ability to choose and use private health insurance. J. Health Commun. 19, 225–239. doi: 10.1080/10810730.2014.936568, PMID: 25315595 PMC4200586

[ref38] Pangestu S. Rencz F. (2023). Comprehensive score for financial toxicity and health-related quality of life in patients with Cancer and survivors: a systematic review and Meta-analysis. Value Health 26, 300–316. doi: 10.1016/j.jval.2022.07.017, PMID: 36064514

[ref39] Patient Protection and Affordable Care Act (2010). Public law 111, 759–762.

[ref40] Rieke K. Schmid K. K. Lydiatt W. Houfek J. Boilesen E. Watanabe-Galloway S. (2017). Depression and survival in head and neck cancer patients. Oral Oncol. 65, 76–82. doi: 10.1016/j.oraloncology.2016.12.014 28109472 PMC8201663

[ref41] Ritchie J Spencer L Bryman A Burgess B. Analyzing Qualitative Data. Chapter 9: Qualitative Data Analysis for Applied Policy Research. London, United Kingdom: Taylor & Francis Group; (1994).

[ref42] Saldaña J. The Coding Manual for Qualitative Researchers, 2nd. Thousand Oaks, CA: SAGE Publications Inc.; (2013).

[ref43] Saunders B. Sim J. Kingstone T. Baker S. Waterfield J. Bartlam B.. (2018). Saturation in qualitative research: exploring its conceptualization and operationalization. Qual. Quant. 52, 1893–1907. doi: 10.1007/s11135-017-0574-8, PMID: 29937585 PMC5993836

[ref44] Sayani A. Dilney J. Kuhnke J. L. McNeil T. (2021). “my Cancer is worth only fifteen weeks?” a critical analysis of the lived experiences of financial toxicity and Cancer in Canada. Int. J. Health Policy Manag. 11, 1814–1822. doi: 10.34172/ijhpm.2021.83, PMID: 34634872 PMC9808211

[ref45] Schaal J. C. Lightfoot A. F. Black K. Z. Stein K. White S. B. Cothern C.. (2016). Community-guided focus group analysis to examine Cancer disparities. Prog Community Health Partnersh 10, 159–167. doi: 10.1353/cpr.2016.0013, PMID: 27018365 PMC4810449

[ref46] Schröder S. L. Schumann N. Fink A. Richter M. (2020). Coping mechanisms for financial toxicity: a qualitative study of cancer patients’ experiences in Germany. Support Care Cancer 28, 1131–1139. doi: 10.1007/s00520-019-04915-w, PMID: 31201545

[ref47] Shih Y. T. Yabroff K. R. Bradley C. J. (2023). Prescription drug provisions in the inflation reduction act: any relief of financial hardship for patients with Cancer? JAMA Oncol. 9, 165–167. doi: 10.1001/jamaoncol.2022.5805, PMID: 36480188 PMC10550564

[ref48] Skrabal Ross X. Gunn K. M. Olver I. (2021). Understanding the strategies rural cancer patients and survivors use to manage financial toxicity and the broader implications on their lives. Support Care Cancer 29, 5487–5496. doi: 10.1007/s00520-021-06086-z, PMID: 33710410

[ref49] Thomson M. D. Siminoff L. A. (2015). Finding medical care for colorectal cancer symptoms: experiences among those facing financial barriers. Health Educ Behav 42, 46–54. doi: 10.1177/1090198114557123 25394821 PMC4604569

[ref50] Timmons A. Gooberman-Hill R. Sharp L. (2013). “It’s at a time in your life when you are most vulnerable”: a qualitative exploration of the financial impact of a cancer diagnosis and implications for financial protection in health. PLoS One 8:e77549. doi: 10.1371/journal.pone.0077549, PMID: 24244279 PMC3823871

[ref51] Tracy S. J. (2010). Qualitative quality: eight “big-tent” criteria for excellent qualitative research. Qual. Inq. 16, 837–851. doi: 10.1177/1077800410383121

[ref52] Tucker-Seeley R. D. (2023). Financial toxicity: a barrier to achieving health equity in cancer care. J. Am. Coll. Radiol. 20, 37–39. doi: 10.1016/j.jacr.2022.12.004, PMID: 36503172 PMC9797364

[ref53] Waters A. R. Mann K. Warner E. L. Vaca Lopez P. L. Kaddas H. K. Ray N.. (2022). “I thought there would be more I understood”: health insurance literacy among adolescent and young adult cancer survivors. Support Care Cancer 30, 4457–4464. doi: 10.1007/s00520-022-06873-2, PMID: 35107600 PMC10512194

[ref54] Weaver K. E. Rowland J. H. Bellizzi K. M. Aziz N. M. (2010). Forgoing medical care because of cost: assessing disparities in healthcare access among cancer survivors living in the United States. Cancer 116, 3493–3504. doi: 10.1002/cncr.25209, PMID: 20549763 PMC3018838

[ref55] Wheeler S. B. Rodriguez-O’Donnell J. Rogers C. Fulcher J. Deal A. Manning M. L.. (2020). Reducing Cancer-related financial toxicity through financial navigation: results from a pilot intervention. Cancer Epidemiol. Biomark. Prev. 29:694. doi: 10.1158/1055-9965.EPI-20-0067

[ref56] Wheeler S. B. Spencer J. C. Pinheiro L. C. Carey L. A. Olshan A. F. Reeder-Hayes K. E. (2018). Financial impact of breast Cancer in Black versus White women. J. Clin. Oncol. 36, 1695–1701. doi: 10.1200/JCO.2017.77.6310, PMID: 29668368 PMC5993169

[ref57] Yezefski T. Steelquist J. Watabayashi K. Sherman D. Shankaran V. (2018). Impact of trained oncology financial navigators on patient out-of-pocket spending. Am. J. Manag. Care 24, S74–S79. PMID: 29620814

[ref58] Zafar S. Y. Abernethy A. P. (2013). Financial toxicity, part I: a new name for a growing problem. Oncology (Williston Park) 27, 80–149. PMID: 23530397 PMC4523887

[ref59] Zhao J. Han X. Zheng Z. Banegas M. P. Ekwueme D. U. Yabroff K. R. (2019). Is health insurance literacy associated with financial hardship among cancer survivors? Findings from a National Sample in the United States. JNCI Cancer Spectrum 3:pkz061. doi: 10.1093/jncics/pkz061 32337486 PMC7050003

[ref60] Zheng Z. Jemal A. Han X. Guy G. P. Jr. Li C. Davidoff A. J.. (2019). Medical financial hardship among cancer survivors in the United States. Cancer 125, 1737–1747. doi: 10.1002/cncr.31913, PMID: 30663039

