# Supplementary material for: Facing financial barriers to healthcare: patient-informed adaptation of a conceptual framework for adults with a history of cancer
Source: Front Psychol. 2023 May 15;14:1178517. doi: 10.3389/fpsyg.2023.1178517 (PMC10225523; doi:10.3389/fpsyg.2023.1178517)
Supplement: Supplementary file 1 [file Data_Sheet_1.docx]

Supplementary Material

Facing financial barriers to healthcare: Patient-informed adaptation of a conceptual framework for adults with a history of cancer

**Caitlin B. Biddell,*^1,2^ Austin R. Waters,^1,2^ Rebekah S.M. Angove,^3^ Kathleen D. Gallagher,^3^ Donald L. Rosenstein,^2,4^ Lisa P. Spees,^1,2^ Erin E. Kent,^1,2,5^ Arrianna Marie Planey,^1,5^ Stephanie B. Wheeler^1,2^**

*** Correspondence:** Caitlin Biddell: cbiddell@live.unc.edu

# Supplemental Appendix 1. Semi-structured interview guide

**Introduction**

1. **Can you briefly tell me about your cancer diagnosis and treatment experience?**
   1. [Follow-up] When were you diagnosed?
   2. [Follow-up] What type of cancer were you diagnosed with? And what stage was your cancer when it was diagnosed?
   3. [Follow-up] Did you have health insurance at the time that you were diagnosed? If so, what type?
   4. [Follow-up] Are you currently undergoing active treatment (including oral chemotherapy)?
   5. [Follow-up] Were you employed at the time of your diagnosis? If so, has your cancer diagnosis and treatment impacted your ability to work?
2. **Prior to your cancer diagnosis, had you ever experienced financial difficulty (i.e., challenges paying for day-to-day necessities)? If so, can you tell me a bit more about what was going on in your life at that time?**

**Experience of Financial Barriers and Impact on Healthcare Decision-making**

1. **We know that cancer is costly, and financial challenges can impact patients in a lot of different ways – from the emotional impact to changes in how patients make decisions about their medical care. You indicated in the survey that you have experienced challenges paying for medical care in the past year** *[IF APPLICABLE (based on screener):* ***and that you stopped or delayed medical care because it cost too much****]***. Can you tell me more about this experience?**
   1. [Probe] What types of care did you have difficulty accessing and/or paying for (including both the cost of the medical care itself, as well as the cost of caregiving, transportation or perhaps lodging to get to this care)? (e.g., chemo or radiation, prescription medications, dental appointments, specialist appointments, other types of aftercare following treatment)
   2. [Probe] Did financial challenges impact your cancer diagnosis experience and how quickly you were able to begin treatment?
   3. [Probe *if Screener Q6=A lot or A little*] Can you talk me through an example of a time when you had to take money from what you would have spent on food, housing, or other basic needs (for you, your family, and any others you care for) to pay for medical care?
   4. [Probe] Have these financial challenges impacted others in your household or community?
   5. [Probe] Have these financial challenges impacted your ability to do things to stay healthy on a daily basis (things like eating healthy, exercising, or anything else you might do to take care of yourself)? How so?
   6. [Probe] Do you feel these financial challenges have impacted how providers (doctors, nurses, etc.) treat you? If so, can you tell me more about that?
   7. [Probe] How has the COVID-19 pandemic impacted your ability to pay for medical care?
2. **How, if at all, have these financial challenges impacted your mental health?**
   1. [Probe] Prior to your cancer diagnosis, had you experienced challenges with your mental health? Had you ever been diagnosed with anxiety or depression?
   2. [Follow-up] How about your overall life satisfaction and the general emotional quality of your everyday experiences?
3. *[If participant indicated ED visit or unexpected hospitalization in the past year in screener]* **You mentioned in the screener questionnaire having an unexpected hospitalization or visit to the emergency room in the past year. We’ve discussed the different financial challenges to care that you have experienced. Do you think this emergency room visit or hospitalization could have been avoided if you had not been experiencing these financial challenges?**
   1. [Follow-up] Was this emergency room visit or hospitalization either directly or indirectly related to your cancer diagnosis?

**Strategies Used to Overcome Financial Barriers to Care**

1. **In the face of these financial challenges you have described, what types of things have you done to try to get the care you need?**
   1. [Probe] One example would be getting connected to the Patient Advocate Foundation. Can you start by telling me how you got connected to PAF and what types of resources you received?
   2. [Probe] Have you applied for/received resources from any other non-profits or applied for or received financial assistance/charity care from the hospital?
   3. [Probe] Have you changed health insurance plan or applied for/received government social assistance (things like food stamps, social security disability insurance, supplemental security income)?
   4. [Probe] Have you used credit cards or taken on medical debt?
2. **Can you tell me about any challenges you faced in finding and accessing financial resources to get needed medical care?**
   1. [Probe] Is there a time that you remember feeling discouraged, overwhelmed, or like you were not able to pursue your goals in this process? Can you tell me about what might have led to that feeling?
   2. [Probe *if participant indicated impact of financial challenges on mental health or emotional well-being in #4 above*] You mentioned earlier that the financial challenges we have been discussing had a negative impact on your mental and emotional health. How, if at all, did this impact your ability to overcome financial barriers to care?
   3. [Probe] Did you feel you had to advocate for yourself or navigate resources on your own? Did you have anyone helping you to find resources? Maybe a healthcare employee, friend, or family member?
   4. [Probe] Did you feel that your interactions with healthcare providers influenced your ability to get connected to resources?
   5. [Probe] What do you think would have lessened the experience of some of these challenges?
   6. [Probe] What types of things did you find to be most helpful in this process of finding resources?
   7. [Probe] What was your greatest motivation to continue finding and applying for resources to overcome financial barriers to care?
   8. [If participant reported experiencing financial difficulties in the past] How do you think your experience with financial challenges in the past influenced your ability to find resources?

**Conclusion**

1. **Is there anything else you would like to share with us about your experience with financial hardship and financial barriers to care related to your cancer diagnosis and treatment?**

Thank you so much for your participation. We really appreciate you taking the time to talk with us.

# Supplemental Appendix 2. Codebook

| **Code (>>>Sub-Code)** | **Decision Rules** |
| --- | --- |
| Clinical Context | Note: Most data will be extracted separate from coding. |
| Current active treatment | Apply for positive and negative responses to the question “Are you currently undergoing active treatment?”, including descriptions of active treatment among interviewees answering affirmatively |
| Current surveillance/monitoring | References to any routine surveillance or monitoring (e.g., scans, blood work) respondent is currently undergoing |
| Pre-diagnosis mental health | References to whether respondent experienced challenges with mental health (e.g., anxiety, depression) prior to their cancer diagnosis |
| Financial Context |  |
| Employment impact | Among employed interviewees, code references to the impact of their diagnosis on ability to work |
| >>>Comfort being open about diagnosis with employer | References to interviewee’s conversations with employer (or lack of) about diagnosis |
| Changes in treatment location due to cost | Code when interviewee describes changing where they sought oncology care for cost- or insurance-related reasons |
| Perceived influence of insurance coverage/ability to pay on quality of care | References to how interviewee perceives their insurance coverage (or lack of) and ability to pay influences their access to and quality of care |
| Financial difficulty prior to cancer diagnosis | References to respondent’s history with financial difficulty prior to their diagnosis – code only one of the below mutually exclusive subcodes |
| >>>Yes, at the time of diagnosis | May also include financial difficulty in the past |
| >>>Yes, in the past | Prior financial difficulty but not at the time of diagnosis |
| >>>No | No financial difficulty before cancer diagnosis |
| Causes of Financial Barriers |  |
| Out-of-pocket medical bills and insurance premiums | References to out-of-pocket medical costs and insurance premiums |
| >>>Unexpected bills | Code when interviewee describes owing money for unexpected out-of-pocket medical costs (whether due to surprise billing, health insurance literacy challenges, etc.) |
| Non-medical costs | References to challenges getting to medical appointments (i.e., paying for or coordinating transportation), paying for food/lodging at appointments, and other downstream expenses resulting from cancer care (e.g., having to buy new clothes) |
| Lost income | References to the financial impact of lost income from time away from work (for patient and/or caregiver); include references to not being able to pay for day to day necessities due to the lost income |
| Covid-19 | References to the role of the pandemic and associated economic impacts in exacerbating (or lessening) the experience of financial hardship, including explanations that it did not affect interviewee’s financial hardship |
| >>>Inflation | References to the current (Fall 2022) inflation in groceries, gas prices, etc. |
| Cost-related delayed or forgone care |  |
| Time to diagnosis and treatment initiation | References to the impact of financial barriers on time to cancer diagnosis, treatment initiation or other aspects of the diagnosis experience |
| >>>Yes | Code instances of delayed or forgone care |
| >>>No | Code instances in which this type of care was received in a timely manner |
| Treatment decision-making and adherence | References to the impact of financial barriers on whether or not to pursue a type of cancer treatment and ability to adhere to it (including the decision to discontinue) |
| >>>Yes | Code instances of delayed or forgone care |
| >>>No | Code instances in which this type of care was received in a timely manner |
| Supportive care | References to not taking all prescribed supportive medications (e.g., for pain, constipation, nausea, etc.); not seeking out or following through on recommended supportive therapies (e.g., physical therapy, counseling); or not being able to pursue alternative medicine approaches (e.g., acupuncture, naturopathic doctors) due to financial barriers |
| >>>Yes | Code instances of delayed or forgone care |
| >>>No | Code instances in which this type of care was received in a timely manner |
| Surveillance/monitoring | References to not receiving or delaying the appropriate follow-up scans, imaging, or exams due to financial barriers |
| >>>Yes | Code instances of delayed or forgone care |
| >>>No | Code instances in which this type of care was received in a timely manner |
| Non-cancer-related medical care | References to medical care needed but not received or delayed for other conditions not directly related to the cancer |
| >>>Yes | Code instances of delayed or forgone care |
| >>>No | Code instances in which this type of care was received in a timely manner |
| Lifestyle Impacts due to Financial Hardship |  |
| Housing-related changes | References to having to move or refinance mortgage due to cancer-related financial hardship; also include challenges affording home repairs and other home upkeep (e.g., cleaning, yardwork) |
| Transportation-related changes | References to having to change mode of transportation due to cancer-related financial hardship (e.g., having to sell a car) |
| Ability to afford eating healthy and exercising | References to the extent to which cancer-related financial hardship has influenced ability to eat healthy and/or organic (including taking recommended vitamins and supplements) and/or ability to exercise or do recreational activities (e.g., afford gym membership) |
| Ability to afford socializing | References to the extent to which cancer-related financial hardship has influenced ability to afford to go out with friends, maintain relationships, etc. |
| “Nothing extra” | References to limiting all household spending to the bare minimum to get by; distinguishing needs vs. wants |
| Impact on family/other members of the household | References to the impact of financial strain on others in the interviewee’s household (e.g., partner, children, parents) |
| >>>Concerns about passing on debt to family members | References to interviewee worry about passing on medical debt to family members or partners |
| Prioritization of healthcare and household necessities | Discussion of how interviewee prioritizes different expenses, including different types of healthcare, household bills, taking care of children/other family members, etc. (including what interviewee is/is not willing to sacrifice) |
| Adverse Events (ED Visit or Unexpected Hospitalization) | Note: Only asked to interviewees who reported an ED visit or hospitalization in the screener and described delaying/forgoing care due to cost |
| Related to financial barriers? | References to whether respondent thinks visit could have been avoided in the absence of financial challenges |
| Resources Used to Overcome Financial Barriers | Include commentary on the lack of, or challenges with, resources within each category |
| Health Insurance | References to changes in health insurance following cancer diagnosis and/or the extent to which health insurance was able to mitigate cost of treatment |
| >>>Private | Including employer-sponsored and marketplace plans |
| >>>Medicare | Including those eligible through disability or age; including Medicare Advantage or supplemental plans, and references to QMB program and the Part D low-income subsidy |
| >>>Medicaid | References to having Medicaid coverage or not qualifying due to eligibility limitations |
| >>>VA/Tricare/Other | Code for health coverage through the VA, Tricare, or any other plan types not captured above |
| >>>Uninsured | References to not having insurance |
| >>>Insurance denials and coverage limitations | References to financial challenges caused by insurance limitations, including the time and energy required to go back and forth with insurance, and commentary on coverage determinations feeling random or illogical |
| Employer-offered Benefits | References to paid time off, Family Medical Leave Act, short-term disability, or other employment-related benefits (excluding employer-sponsored health insurance, which is captured above) |
| Government assistance | References to governmental social assistance programs |
| >>>Disability | Including commentary on limitations/benefits of disability and process of applying for it |
| >>>SNAP | References to receiving food stamps or not qualifying due to eligibility limitations |
| Hospital-based financial assistance | References to “charity care”, hospital write-offs (or lack of), payment plans, and any hospital foundation assistance (e.g., hotels, gas cards) |
| Pharmaceutical medication assistance | References to medications being covered by grants from pharmaceutical companies or pharmacy discounts |
| Non-profit organizations | References to funding from non-profit organizations, including commentary on the extent to which available resources are sufficient |
| >>>Reliance on resources that are not guaranteed | Commentary on the nature of non-profit resources in that they are not guaranteed and may run out at any point |
| Financial support from family/community | References to community fundraisers, GoFundMe’s, direct financial support, etc. |
| Filter: Protective factors | Factors that minimize the negative impact of financial barriers |
| Familiarity with financial difficulties | References to how experiencing financial difficulty prior to cancer diagnosis helped or hindered ability to overcome financial barriers |
| Motivation | References to what motivates interviewee to continue pursuing resources to overcome financial barriers |
| >>>Intrinsic | Willingness to prioritize healthcare above all else to survive and keep going on a daily basis |
| >>>Family/children/community | Wanting to be around for and/or provide for community |
| Self-advocacy and ability to navigate resources | References to interviewee speaking up to ensure they got the resources and care they needed; extent to which interviewee has been able to navigate resources on their own, including challenges in doing so, feeling helpless, etc. |
| >>>Comfort asking for help | Extent to which interviewee feels comfortable asking for help and/or expressing concerns about cost to care team, family members, etc.; include references to feeling like a burden |
| >>>Independent research | References to interviewee independently searching online or elsewhere for resources |
| >>>Sharing resources and helping others | References to interviewee enjoying sharing resources they find with others |
| Filter: Modifying Factors | Factors that have the potential to be either protective or predisposing |
| Care team interactions | References to positive or negative interactions with the care team related to cost and the ability to afford care |
| >>>Navigator/social worker/patient advocate | References to interacting with individual at the hospital system whose primary responsibility it is to help address patient financial and psychosocial concerns |
| >>>Care team-initiated cost conversations? Yes | Use this code when care team initiates cost conversations with interviewee, including references cost transparency at the facility |
| >>>Care team-initiated cost conversations? No | References to members of the care team not initiating conversations about cost with the interviewee, including references to interviewee feeling like this is not the responsibility of the care team or wishing they had brought cost up in care discussions |
| Coping Strategies | References to how interviewee typically responds to financial stress |
| >>>Adaptive | Problem-focused strategies (e.g., facing problems head on, bringing cost challenges up to care team, searching for resources) |
| >>>Maladaptive | Emotion-focused strategies (e.g., avoidance) |
| Individual responsibility | References to having personal, family, or professional obligations that either contributed to the stress of financial barriers or helped interviewee cope with barrier |
| Social support/isolation | References to either feeling supported by community/family (including online communities) or feeling alone in the process of dealing with financial barriers (e.g., help finding/applying for resources, managing bills) |
| Filter: Predisposing Factors | Factors that may amplify negative repercussions of financial barriers |
| Perceived injustice/discrimination | References to feeling prejudice, judgment, or discrimination from health system, providers, government social services, etc. |
| Mental and emotional health challenges | References to cost-related worry, overwhelm, anxiety, and/or depression; Also include references to how mental health challenges and emotional well-being decrements influence interviewee’s ability to find resources and overcome financial barriers |
| >>>Lost independence/purpose | References to feeling stifled in life, career, relationships, etc. |
| >>>Concerns about the future | References to “What if?” scenarios, not seeing a path forward, etc. |
| >>>Impact of stress on health | References to the perceived impact of financial stress on health |
| Physical health challenges | References to chronic pain, fatigue, brain fog, or other effects of cancer treatment making it difficult to overcome financial barriers |
| Lens | Influence of attitude, personality, or worldview on approach to dealing with financial barriers |
| Positive outlook | References to trying to have a positive outlook/worldview/attitude despite negative circumstances |
| Religiosity | References to religion or spirituality in relation to coping with emotional impact of financial barriers |
| Assumption/belief that I will get the care I need | References to relying on a baseline belief that interviewee will get the necessary care one way or another |
| Negative outlook | References to feeling negatively about money and the financial barriers faced |
| Resiliency |  |
| Determination vs. despair | References to how respondents’ have adapted to experiencing financial barriers), ranging from determination to despair; how past experiences have shaped their approach to current and future hardships |
| Recommendations to lessen the experience of financial barriers | Code responses to the question, “What would have lessened the financial challenges you experienced?” |
| Policy | Recommendations made related to national, state, or local laws and regulations; insurance system structure and eligibility; health system structure (broadly, not the specific hospital) |
| Organizational – Health System | Recommendations made related to health system practices and policies (including adding additional positions or job descriptions) |
| Organizational – Other | Recommendations made related to the role of non-profit organizations (or other organizations) in ameliorating financial concerns |
| Individual | Recommendations made related to how individuals can personally prepare for and deal with financial barriers |
